# Optimizing n-3 PUFA dietary enrichment in slow-growing Korat chickens using lipid-based nanoparticles: Effects on growth performance, carcass traits, meat quality, meat fatty acid composition, and blood biochemical parameters

**DOI:** 10.1016/j.psj.2025.104982

**Published:** 2025-03-06

**Authors:** Piyaradtana Homyok, Wunpen Sonsamrong, Nitipol Chainet, Wichuta Khosinklang, Anyanee Kamkaew, Teerapong Yata, Elisabeth Baéza, Cécile Berri, Amonrat Molee, Wittawat Molee

**Affiliations:** a School of Animal Technology and Innovation, Institute of Agricultural Technology, Suranaree University of Technology, Nakhon Ratchasima, 30000, Thailand; b School of Chemistry, Institute of Science, Suranaree University of Technology, Nakhon Ratchasima 30000, Thailand; c Biochemistry Unit, Department of Physiology, Faculty of Veterinary Science, Chulalongkorn University, Bangkok 10330, Thailand; d INRAE, Université de Tours, UMR BOA, 37380 Nouzilly, France

**Keywords:** n-3 PUFA, Lipid-based nanoparticle, Slow-growing chicken, Meat quality, Health status

## Abstract

This research aimed to investigate the optimization of lipid-based nanoparticles to improve the utilization of n-3 PUFA source in chicken diets. Three groups of slow-growing Korat chickens were reared under the same conditions and fed a diet containing 6 % rice bran oil (**RBO**, control group), 3 % tuna oil (**3 % TO**) and 3 % tuna oil in targeted lipid-based nanoparticles (**3 % TO-TNP**). The growth performance, carcass composition, meat quality, fatty acid profile of breast and thigh meat, hematological and plasma biochemical parameters were evaluated. The dietary lipid source had no effect on the growth performance, carcass composition, or breast and thigh meat quality (*P* > 0.05). Compared to the control group, the 3 % TO and 3 % TO-TNP groups exhibited lower levels of C18:2n-6 (linoleic acid) and n-6 PUFA (*P* < 0.05). There was no significant difference between groups in the C18:3n-3 (**ALA**) level in breast and thigh meat, whereas the levels of C20:5n-3 (**EPA**) and C22:6n-3 (**DHA**) were higher in both TO groups. However, due to the high temperature required during the drying step of the nanoparticle synthesis process, n-3 PUFA enrichment was more efficient when TO was directly incorporated into the diet than when using targeted nanoparticles. Nevertheless, there was no difference between the 3 % TO and 3 % TO-TNP groups in the n-6/n-3 ratio (*P* > 0.05). The hemoglobin and hematocrit values were higher in 3 % TO-TNP than in the other groups (*P* < 0.05). In the plasma, HDL level was reduced in the TO groups compared to the control group (*P* < 0.05) suggesting a lower cholesterol hepatic synthesis and export. The TO groups had a higher activity of alanine amino transferase but a lower creatinine content than the control group (*P* < 0.05). The uric acid content was higher in 3 % TO-TNP group than in the 3 % TO group (*P* < 0.05) but no different compared with the control group. However, the values obtained for the hematological and biochemistry parameters measured in blood and plasma were within the normal range for chickens. To conclude, the supplementation of lipid-based nanoparticles allowed for change the fatty acid composition of chicken meat without any negative effect on production and health status of chickens.

## Introduction

In recent decades, there has been increasing interest for functional foods for human health, such as the enrichment of meat products with n-3 polyunsaturated fatty acids (**PUFA**), which can be achieved through the supplementation of animal feed with n-3 oil sources. However, n-3 oil sources present numerous challenges, such as an unpleasant taste and aroma, low storage stability, and reduced bioavailability of important PUFA like eicosapentaenoic acid (**EPA**) and docosahexaenoic acid (**DHA**). Enhancing the stability of n-3 PUFA using lipid-based nanoparticles could improve the utilization of these n-3 PUFA sources.

Korat chicken is a crossbreed between a male Thai indigenous chicken (Lueng Hang Khao) and a female from a breeder line developed at Suranaree University of Technology (**SUT**). This slow-growing chicken can be used to produce functional meat enriched with n-3 PUFA. Previous studies showed that dietary supplementation with 4 % tuna oil increased the n-3 PUFA content in Korat chicken meat (Hang et al., 2018a). Additionally, the literature demonstrated that n-3 PUFA sources are susceptible to lipid oxidation, which affects feed quality (Sanguansri et al., 2013). Although encapsulation can protect these lipids, this approach is insufficient for enhancing the utilization of n-3 PUFA sources, as some of these fatty acids are also distributed in non-targeted organs. To date, research has primarily focused on meat enrichment with n-3 PUFA rather than the large-scale production of feed supplemented with stabilized n-3 PUFA sources.

The incorporation of n-3 PUFA into skeletal muscle occurs in vesicle form. The fatty acids are first transferred to hepatocytes (Niot et al., 2009), a process known as “hepatic first-pass metabolism” (Poonia et al., 2016). According to Keegan et al. (2020), the accumulation of n-3 PUFA primarily occurred in the liver, adhering fat, and skin followed by breast and thigh muscles. Therefore, feed formulation requires higher n-3 PUFA supplementation to achieve the recommended level of meat enrichment with n-3 PUFA. To reduce accumulation in non-target organs, nanotechnology could be a more appropriate technology (Lu et al., 2021). Lipid-based nanoparticles are suitable carriers for improving bioavailability. They are non-toxic to cells (Saedi et al., 2018) and the use of modified surfactant supports gastrointestinal tolerance (Zhang et al., 2011) while allowing direct delivery to skeletal muscles (Yeh et al., 2014). Thus, this technology could provide an opportunity to enhance the precision of bioactive compound utilization and improve the nutritional value of meat.

Several researchers claimed the safety of using lipid-based nanoparticles (Das and Chaudhury, 2011; Zhou et al., 2015; Rodriguez-Ruiz et al., 2018). Moreover, most research on nanotoxicity focused on metals and polymers. However, lipid nanoparticles showed a lower toxicity response due to their biodegradable and harmless properties (Winter et al., 2016). Nonetheless, some studies showed that the material of lipid nanoparticle can affect certain hematological parameters such as a decrease in erythrocytes or immunogenicity (Lim et al., 2012). Therefore, to use nanoparticles appropriately level, it is important to study their safety to develop feed additives and determine the level of supplementation that does not induce toxic effects.

Therefore, this research study aimed to generate lipid-based nanoparticles that could reduce the utilization of n-3 oil sources in feed formulation and enhance the efficiency of n-3 PUFA accumulation in slow-growing Korat chicken meat. The effects of n-3 PUFA-loaded lipid-based nanoparticles in diet formulation will be investigated on growth performance, carcass and meat quality, fatty acid composition of muscles, and hematological and biochemical parameters in blood of Korat chickens. Finally, the experimental results will be analyzed and discussed to optimize the production of nanocarriers.

## Materials and methods

### Preparation of targeted lipid nanoparticles

Tuna oil loaded lipid-based nanoparticles were prepared by hot and high-pressure homogenization approach (Yostawonkul et al., 2017). Briefly, sorbitan oleate, alkyl polyglucoside (Croda, Bangkok, Thailand) and crude tuna oil (Nutritech Co. Ltd, Samut Sakhon, Thailand) were blended and melted above 70 °C to form an organic phase. Meanwhile, the dispersing emulsifying agent was made up of glycerol, Polysorbate 20, Poloxamer 188 (Croda, Bangkok, Thailand) in distilled water. The aqueous phase was gradually added to the lipid phase with continuous stirring for 3 min, leading to the formation of an initial emulsion. The pre-emulsion underwent homogenization utilizing a high-speed T25 digital homogenizer (IKA Works, Inc., Staufen, Germany) for 3 min and sonication by an ultrasonic homogenizer (VC 750, Vibra-Cell™, Sonics & Materials Inc., Newtown, CT) for 5 min, resulting in the production of targeted lipid-based nanoparticles loaded with tuna oil. Subsequently, the dispersion underwent cooling in an ice water bath. The prepared dispersion was stored in an airtight container and maintained at 4 °C. Subsequently, the sample was dried using a hot air oven set at 80 °C for 10 h.

### Birds and housing

All procedures in the present study were approved by the Ethics Committee on Animal Use of the Suranaree University of technology, Nakhon Ratchasima, Thailand (SUT-IACUC-002/2022). The experiment was conducted at SUT farm. One-day old female Korat chickens were reared on floor pens with a density of 8 birds/m^2^. The birds were maintained at a brooding temperature of 30 °C to 35 °C during the first 21 d of age. Then, they were housed in an open-sided facility with natural ventilation, exposed to ambient conditions. The temperature ranged from 14.1 °C to 32.2 °C, with an average relative humidity of 70.68 % (National Agricultural Big Data Center, Nakhon Ratchasima, Thailand). They were vaccinated against Marek's disease at the hatchery, followed by Newcastle disease and infectious bronchitis at 7 and 21 d of age. Additionally, a Gumboro disease was administered at 14 d of age. The chicks were fed a corn-soybean meal-based diet containing 21 % CP and 3,000 kcal ME/kg from d 1 to 21, and 19 % CP and 3,100 kcal ME/kg from d 22-49. The chickens were reared together from hatching until 49 d of age, reaching an average body weight (**BW**) of 903.14 ± 17.71 g.

### Experimental design

The experimental model followed a completely randomized design, in which 49-d-old female Korat chickens were randomly allocated into three dietary treatments groups. Each treatment consisted of five replications, with 25 birds per replication. The experimental chickens were maintained in an open indoor housing with the temperature ranged from 12.6 °C to 35.1 °C, with an average relative humidity of 69.60 % (National Agricultural Big Data Center, Nakhon Ratchasima, Thailand). An automatic ventilation fan maintained a temperature range of 30 °C to 35 °C within the housing. A basal diet was formulated based on corn-soybean containing 6 % of rice bran oil (Thai Ruam Jai Korat Co., Ltd., Nakhon Ratchasima, Thailand) (**Control group**). In the other dietary treatments, 3 % of the RBO content was replaced with 3 % tuna oil (**3 % TO**) or 3 % tuna oil in targeted lipid-based nanoparticles (**3 % TO-TNP**). All experimental diets were formulated to provide 3,180 kcal/kg ME and 19 % CP for the finisher (d 49 to 70) (Table 1). Feed-grade oils were used in all diets. The birds received the diet and drinking water ad libitum.

**Table 1:** Ingredients and nutrient composition of the experimental diets (as-fed basis).

	49 to 70 days		
Ingredients	Control	3 % TO	3 % TO-TNP
Corn	58.10	58.10	58.10
Soybean meal 44 %	32.42	32.42	32.42
Tuna oil	-	3.00	-
Tuna oil TNP	-	-	3.00
Rice bran oil	6.00	3.00	3.00
DL-methionine	0.16	0.16	0.16
Salt	0.35	0.35	0.35
CaCo_3_	1.27	1.27	1.27
P21 (monocalcium phosphate)	1.20	1.20	1.20
Premix^1^	0.50	0.50	0.50
Total (%)	100.00	100.00	100.00
Calculated nutrients			
Metabolizable energy (kcal/kg)	3180.00	3180.00	3180.00
Crude proteins	18.80	18.80	18.80
Crude fiber	3.72	3.72	3.72
Crude fat	8.47	8.47	8.47
Calcium	0.80	0.80	0.80
Total phosphorus	0.64	0.64	0.64
Available phosphorus	0.40	0.40	0.40
Lysine	0.94	0.94	0.94
Methionine	0.45	0.45	0.45
Analyzed nutrient levels (%)^2^			
Total lipids	6.81	6.76	6.51
C18:2n-6, Linoleic acid	35.88	27.51	31.59
C18:3n-3, ALA	0.50	0.95	0.87
C20:5n-3, EPA	0.00	2.83	1.19
C22:6n-3, DHA	0.00	8.66	3.11
SFA	24.86	26.04	27.14
MUFA	38.70	32.55	35.11
PUFA	36.44	41.41	37.75
n-3 PUFA	0.50	12.44	5.16
n-6 PUFA	35.94	28.97	32.58
n-6/n-3 ratio	72.26	2.33	6.31

1 Premix contained the following nutrients (units are expressed per kg of diet): vitamin A, 15,000 IU; vitamin D3, 3,000 IU; vitamin E, 25 IU; vitamin K3, 5 mg; vitamin B1, 2 mg; vitamin B2, 7 mg; vitamin B6, 4 mg; vitamin B12, 25 mg; pantothenic acid, 11.04 mg; nicotinic acid, 35 mg; folic acid, 1 mg; biotin, 15 µg; choline chloride, 250 mg; Cu, 1.6 mg; Mn, 60 mg; Zn, 45 mg; Fe, 80 mg; I, 0.4 mg; Se, 0.15 mg.

2 ALA, Alpha-linolenic acid; EPA, Eicosapentaenoic acid; DHA, Docosahexaenoic acid; SFA, Saturated fatty acids; MUFA, Monounsaturated fatty acids; PUFA, Polyunsaturated fatty acid.

### Data and sample collection

#### Growth performance

The chickens were weighed, and the feed intake (**FI**) was recorded weekly to calculate the average daily gain (**ADG**) and feed conversion ratio (**FCR**) of each group. The mortality rate was also recorded.

### Carcass and meat quality

#### Slaughter procedure and carcass measurements

At 70 d of age, 60 chickens (4 females per replicate x 5 replicates per treatment x 3 groups) randomly chosen were slaughtered after 12 h feed withdrawal period. The chickens were transported to the slaughterhouse within 1 h and then rested for about 30 min. All chickens were stunned by electricity (current parameters, V and Hz), scalding at 60 °C, then de-feathering by machine and eviscerating manually. They were washed with clean water and put in a chilled room (4 °C, 24 h), before cutting into portions. The hot carcass (without viscera, head, neck, feet, and shanks), abdominal fat (considered as the fat extending within the ischium, surrounding the cloaca, and adjacent to the abdominal muscle), and edible giblets were weighted. The weight of abdominal fat and edible giblets were then calculated as the proportion of the hot carcass of the chicken. The portions of carcass after chilled including breast and thigh meats were expressed as the percentage of the chilled carcass weight.

### Meat quality

The pH and meat color of the breast and thigh meats were measured before being stored at -20 °C for subsequent evaluation of meat quality, including drip loss, cooking loss, and shear force measurement.

### pH measurement, drip loss and cooking loss

The pH value was measured at 24 h post-mortem in the breast and thigh muscle at a depth of 0.5 to 1.0 cm. The breast and thigh fillet pH were measured by inserting a probe into the muscle and using a pH meter (Testo 206-pH2, Testo SE & Co. KGaA, Titisee-Neustadt, Germany) with a precision of 0.01 pH unit.

After chilling for 24 h, the water holding capacity was determined as drip loss (hung as airtight container, chilled for 72 h at 4 °C) and cooking loss (boiled in a water bath in open plastic bags until as internal temperature of 85 °C was reached). The drip loss and cooking loss were expressed as a percentage of the initial weight.

$$\%\;\text{Drip}\;\text{loss}=\left(\frac{\mathrm{weight}\;\text{before}\;\text{storage}-\mathrm{weight}\;\text{after}\;\text{storage}}{\mathrm{weight}\;\mathrm{before}\;\mathrm{storage}}\right)\times 100$$

$$\%\;\text{Cooking}\;\text{loss}=\left(\frac{\mathrm{weight}\;\text{before}\;\text{cooking}-\mathrm{weight}\;\text{after}\;\text{cooking}}{\mathrm{weight}\;\mathrm{before}\;\mathrm{cooking}}\right)\times 100$$

### Meat color measurement

The meat color was measured using a Hunter Lab ColorQuest XE spectrophotometer, with QC Software (Reston, VA) calibrated against black and white reference tiles. L* (lightness), a* (redness), and b* (yellow) values were obtained by an illuminant/observer D65/10°. The measurements were conducted through the transparent packaging material. Three random readings were taken, and subsequently averaged on a sample surface for the purpose of statistical analysis.

### Shear force measurement

Cooked meat samples were used to determine shear force value. At least 3 subsamples will be removed (2.0 × 1.0 × 0.5 cm) by cutting parallel to the muscle fibers from each cooked sample. A Texture Analyzer (TA-XT2, Texture Technologies Corp., Scarsdale, NY) equipped with a Warner-Bratzler shear force apparatus was used to determine the peak shear force values of subsamples. The crosshead speed was set at 20 cm/min. Peak WBSF values will be determined by averaging the values of three subsamples (Tasoniero et al., 2016).

### Fatty acid composition

Samples of breast and thigh meat were collected from 10 females per group and stored at -20°C until analysis fatty acid profiles.

The lipids were extracted from approximately 5 g of each sample using 90 ml of chloroform:methanol (2:1, v/v) (Folch et al., 1957). Then, 20 to 25 mg of extracted fat were methylated (Metcalfe et al., 1966). The fatty acid methyl esters (FAME) were analyzed using gas chromatography (Hewlett-Packard 7890A; Agilent Technologies, Santa Clara, CA) with a capillary column (SP 2560, Supelco Inc., Bellefonte, PA, USA, 100 m × 0.25 mm i.d., 0.20 µm film thickness) and a flame ionization detector. The carrier gas was helium at a flow rate of 0.95 ml/min. The temperatures of the injector and detector were 260 °C. The initial column temperature was 70 °C, which was then raised to 175 °C at a rate of 13 °C/min, and finally raised to 240 °C at a rate of 4 °C/min. The identified and quantified of compound were performed by using Masshunter software (v10.0.707.0, Agilent, USA) (Zhang et al., 2022).

### Blood parameters

#### Blood sample collection

At 70 d of age, before the feed withdrawal period, blood samples were collected on 10 chickens per group at jugular and cutaneous ulnar veins. Blood samples were kept into lithium heparin tubes for haematological analysis and ethyl diamine tetra acetic acid (**EDTA**) tube for further analysis of plasma biochemical parameters. All blood parameter analysis were measured by a veterinary laboratory (Vet Central Lab, Nakhon Ratchasima, Thailand).

Heparinized whole blood samples were used to determine red blood cell (**RBC**), haemoglobin (**Hb**), packed cell volume (**PCV**), mean corpuscular volume (**MCV**), mean corpuscular haemoglobin (**MCH**), mean corpuscular haemoglobin concentration (**MCHC**), white blood cells (**WBC**). Heterophils, Basophils, Eosinophil, Lymphocyte, Monocytes according to Abdulazeez et al. (2016).

The value of Hb, RBC and PCV were used to calculate MCV [(PCV/RBC) × 10], MCHC [(Hb/PCV) × 100] (Davies et al., 1984) and MCH [(Hb/RBC) × 10] (Jain, 1986).

Plasma samples were used to quantify concentrations of clinical chemistry biomarkers including glucose, cholesterol, triglycerides, low density lipoprotein (**LDL**), high density lipoprotein (**HDL**), total proteins, albumin, total bilirubin, direct bilirubin, globulin, blood urea nitrogen, creatine phosphokinase (**CPK**), lactate dehydrogenase (**LDH**), alanine amino transferase (**ALT**), aspartate amino transferase (**AST**), creatinine, Uric acid, sodium (**Na**), potassium (**K**), chloride (**Cl**), magnesium (**Mg**) (Akinola and Etuk, 2015).

### Statistical Analysis

Analysis of variance will be performed by GLM procedure for a completely randomized design using SPSS Version 18.0 (SPSS Inc., Chicago, I11., USA). University Edition with the following statistical model:

yij=μ+τi+εij,

where yij = the dependent variable, μ = the overall mean, τi = the treatment effect, and εij = the random residual error. Significant differences between treatment means were assessed by Tukey's multiple comparison tests after a significant F-test. The level of statistically significance will establish at *P* < 0.05. All values were expressed as means ± standard error of the mean (**SEM**), which represents the pooled SEM for the model.

## Results

### Fatty acid composition of rice bran oil, tuna oil, and lipid-based nanoparticles

The fatty acid profiles of the oils used for supplementation are shown in Table 2. Rice bran oil was primarily rich in C18:1n-9 and C18:2n-6, with a notably high n-6/n-3 ratio. Tuna oil contained higher levels of n-3 PUFAs, particularly EPA and DHA, resulting in a low n-6/n-3 ratio. The lipid-based nanoparticles exhibited relatively high levels of n-6 PUFAs (C18:2n-6 and C20:4n-6), along with moderate amounts of EPA and DHA, leading to a higher n-6/n-3 ratio compared to tuna oil.

**Table 2:** Fatty acid composition of rice bran oil, tuna oil and lipid-based nanoparticles used in the diets.

Fatty acids (g/100 g total FA)^1^	Rice bran oil	Tuna oil	Tuna oil in lipid-based nanoparticles
C14:0, Myristic acid	0.45	4.65	6.06
C15:0, Pentadecanoic acid	-	16.57	-
C16:0, Palmitic acid	21.75	20.28	29.43
C18:0, Stearic acid	2.36	5.28	8.46
C16:1, Palmitoleic acid	0.21	4.73	5.93
C24:1, Nervonic acid	-	0.28	0.25
C18:1n-9, Oleic acid	41.48	14.16	22.64
C18:2n-6, Linoleic acid	33.13	2.84	7.69
C20:4n-6, Arachidonic acid	0.05	2.52	4.30
C18:3n-3, ALA	0.57	1.68	2.40
C20:5n-3, EPA	-	6.98	3.60
C22:6n-3, DHA	-	20.03	9.24
SFA	24.56	46.78	43.95
MUFA	41.69	19.17	28.82
PUFA	33.75	34.05	27.23
n-3 PUFA	0.57	28.69	15.24
n-6 PUFA	33.18	5.36	11.99
n-6/n-3 ratio	58.09	0.19	0.79

1 ALA, Alpha-linolenic acid; EPA, Eicosapentaenoic acid; DHA, Docosahexaenoic acid; SFA, Saturated fatty acids; MUFA, Monounsaturated fatty acids; PUFA, Polyunsaturated fatty acid.

### Growth performance

The dietary treatments had no effect (*P* > 0.05) on the growth performance and mortality rate of chickens as shown in Table 3.

**Table 3:** Growth performance of Korat chickens fed diets containing rice bran oil (control), tuna oil (3 % TO) or tuna oil in lipid-based nanoparticles (3 % TO-TNP)^1^.

	Treatments^2^				
Item^3^	Control	3 % TO	3 % TO-TNP	SEM	P-value
Initial BW (d 49)	894.61	908.00	906.80	4.57	0.449
Final BW (d 70)	1342.00	1333.20	1344.02	5.95	0.761
BWG	447.39	425.20	437.22	6.69	0.430
FI	1624.24	1593.82	1681.35	23.25	0.318
FCR	3.64	3.76	3.85	0.07	0.487
Mortality rate	0.00	0.00	1.67	0.38	0.110

1 Each mean represents values from 125 chickens per group (5 replicates × 25 chickens/ replicate).

2 Treatment consisted of 6% rice bran oil (control), 3% rice bran oil + 3% tuna oil (3% TO), 3% rice bran oil + 3% tuna oil in lipid-based nanoparticles (3% TO-TNP).

3 BW, body weight; BWG, body weight gain; FI, feed intake; FCR, feed conversion ratio; SEM, Standard error of the mean.

### Carcass and meat quality

#### Carcass composition

The carcass composition of Korat chickens is detailed in Table 4. The 3 % TO group had a higher dressing percentage than the control group (*P* < 0.05). Both TO groups had higher percentages of gizzard than the control group (*P* < 0.05). For all the other cut parts, there were no significant differences between groups (*P* > 0.05).

**Table 4:** Carcass composition of Korat chickens fed diets containing rice bran oil (control), tuna oil (3 % TO) or tuna oil in lipid-based nanoparticles (3 % TO-TNP) and slaughtered at 70 days of age^1^.

	Treatments^2^				
Items	Control	3 % TO	3 % TO-TNP	SEM	P-value
Live weight (g)	1389.89	1379.87	1351.22	3.24	0.087
Hot carcass weight (g)	903.40	913.47	889.71	4.25	0.071
Chilled carcass weight (g)	899.01	906.39	885.35	3.87	0.067
Dressing^3^	65.01^a^	66.18^b^	65.85^ab^	0.19	0.021
% of hot carcass weight					
Heart	0.60	0.53	0.60	0.01	0.084
Spleen	0.48	0.43	0.50	0.04	0.761
Liver	2.47	2.39	2.58	0.04	0.245
Gizzard	2.20^b^	2.58^a^	2.56^a^	0.07	0.034
Abdominal fat	1.97	1.27	1.82	0.14	0.104
% of chilled carcass weight					
Breast meat	18.93	18.69	18.96	0.17	0.795
Thigh meat	14.84	14.48	14.21	0.17	0.335

a,b Means within row carry on common superscript are significantly different at *P* < 0.05.

1 Carcass composition represents values from 20 chickens per group (5 replicates × 4 chickens/ replicate).

2 Treatment consisted of 6 % rice bran oil (control), 3 % rice bran oil + 3 % tuna oil (3 % TO), 3 % rice bran oil + 3 % tuna oil in lipid-based nanoparticles (3 % TO-TNP).

3 Without viscera, head, neck, feet and shank, calculated as a percentage of live weight.

### Meat quality

The average values of meat quality traits are presented in Table 5. The dietary treatments had no significant effect (*P* > 0.05) on the quality traits measured on breast and thigh meat.

**Table 5:** Meat quality traits of Korat chickens fed diets containing rice bran oil (control), tuna oil (3 % TO) or tuna oil in lipid-based nanoparticles (3 % TO-TNP) and slaughtered at 70 days of age^1^.

	Treatments^2^				
Items	Control	3 % TO	3 % TO-TNP	SEM	P-value
Breast meat					
pH 24 h	5.54	5.53	5.53	0.01	0.832
Drip loss (%)	8.14	7.27	8.52	0.32	0.274
Cooking loss (%)	17.65	17.46	16.66	0.23	0.170
Breast WBS^3^	2.88	3.07	2.50	0.14	0.235
L* (lightness)	52.35	53.40	53.60	0.38	0.376
a* (redness)	5.80	5.12	5.26	0.14	0.126
b* (yellowness)	15.87	16.20	15.96	0.17	0.749
Thigh meat					
pH 24 h	6.13	6.09	6.11	0.03	0.854
Drip loss (%)	5.25	5.71	6.23	0.22	0.192
Cooking loss (%)	19.70	20.13	20.81	0.31	0.371
Thigh WBS^3^	2.83	2.53	2.59	0.07	0.175
L* (lightness)	41.46	41.54	41.26	0.32	0.944
a* (redness)	11.89	11.63	11.89	0.16	0.772
b* (yellowness)	12.17	12.68	12.15	0.22	0.583

1 Each mean represents values from 20 chickens per group (5 replicates × 4 chickens/ replicate).

2 Treatment consisted of 6 % rice bran oil (control), 3 % rice bran oil + 3 % tuna oil (3 % TO), 3 % rice bran oil + 3 % tuna oil in lipid-based nanoparticles (3 % TO-TNP).

3 WBS: Warner-Bratzler shear force expressed as kgf/0.5 cm.^2^

### Lipid content and fatty acid composition

The dietary effect on the fatty acid composition of breast and thigh meat is presented in Table 6, Table 7. The dietary treatment had no effect on the lipid content of breast and thigh meat. In the breast meat, both TO groups had lower levels of C18:2n-6, C20:4n-6, n-6 PUFA and C24:1 but higher levels of EPA, DHA, PUFA and n-3 PUFA than the control group (*P* < 0.06). The n-6/n-3 ratio was 4 to 5-fold lower for the TO groups compared to the control group (*P* < 0.05). The enrichment of breast meat with EPA, DHA and n-3 PUFA was higher in the 3 % TO group than in 3 % TO-TNP group (*P* < 0.06). There were no significant differences between groups for the other fatty acids (*P* > 0.05).

**Table 6:** Total lipids (g/100 g fresh meat) and fatty acid composition (g/100 g total FA) of breast meat of Korat chickens fed diets containing rice bran oil (control), tuna oil (3 % TO) or tuna oil in lipid-based nanoparticles (3 % TO-TNP) and slaughtered at 70 days of age^1^.

	Treatments^2^				
Fatty acid composition^3^	Control	3 % TO	3 % TO-TNP	SEM	P-value
Total lipids	0.85	0.91	0.89	0.02	0.620
C14:0, Myristic acid	0.26	0.41	0.43	0.04	0.153
C15:0, Pentadecanoic acid	4.38	4.64	5.08	0.21	0.425
C16:0, Palmitic acid	22.26	22.05	21.90	0.14	0.625
C18:0, Stearic acid	10.68	10.25	11.27	0.21	0.144
C16:1, Palmitoleic acid	0.59	0.92	0.80	0.07	0.162
C24:1, Nervonic acid	3.11^a^	0.87^b^	1.61^b^	0.28	< 0.001
C18:1n-9, Oleic acid	24.85	23.96	23.76	0.43	0.578
C18:2n-6, Linoleic acid	19.62^a^	17.01^b^	17.56^b^	0.49	0.059
C20:4n-6, Arachidonic acid	11.14^a^	8.23^b^	9.31^b^	0.52	0.053
C18:3n-3, ALA	0.30	0.32	0.40	0.03	0.395
C20:5n-3, EPA	0.23^c^	1.01^a^	0.65^b^	0.10	< 0.001
C22:6n-3, DHA	2.05^c^	10.80^a^	7.21^b^	0.99	< 0.001
SFA	37.58	37.37	38.68	0.33	0.234
MUFA	28.55	25.76	26.18	0.58	0.097
PUFA	33.87^b^	36.88^a^	35.14^a^	0.54	0.059
n-6 PUFA	30.76^a^	25.24^b^	26.88^b^	0.80	0.004
n-3 PUFA	2.47^c^	12.24^a^	8.62^b^	1.09	< 0.001
n-6/n-3 ratio	13.97^a^	2.65^b^	3.42^b^	1.59	< 0.001

a,b,c Means within row carry on common superscript are significantly different at *P* < 0.06.

1 Each mean represents values from 10 chickens per group (5 replicates × 2 chickens/ replicate).

2 Treatment consisted of 6 % rice bran oil (control), 3 % rice bran oil + 3 % tuna oil (3 % TO), 3 % rice bran oil + 3 % tuna oil in lipid-based nanoparticles (3 % TO-TNP).

3 ALA, Alpha-linolenic acid; EPA, Eicosapentaenoic acid; DHA, Docosahexaenoic acid; SFA, Saturated fatty acids; MUFA, Monounsaturated fatty acids; PUFA, Polyunsaturated fatty acid.

**Table 7:** Total lipids (g/100 g fresh meat) and fatty acid composition (g/100 g total FA) of thigh meat of Korat chickens fed diets containing rice bran oil (control), tuna oil (3 % TO) or tuna oil in lipid-based nanoparticles (3 % TO-TNP) and slaughtered at 70 days of age^1^.

	Treatments^2^				
Fatty acid composition^d^	Control	3 % TO	3 % TO-TNP	SEM	P-value
Total lipids	1.30	1.08	1.15	0.58	0.415
C14:0, Myristic acid	0.38^b^	0.64^a^	0.62^a^	0.037	0.001
C15:0, Pentadecanoic acid	2.66	3.77	3.35	0.229	0.133
C16:0, Palmitic acid	22.12	21.92	22.26	0.199	0.814
C18:0, Stearic acid	10.54	11.39	12.16	0.348	0.167
C16:1, Palmitoleic acid	0.94	1.28	1.27	0.094	0.255
C24:1, Nervonic acid	2.37^a^	0.64^b^	1.18^b^	0.213	< 0.001
C18:1n-9, Oleic acid	26.15	21.97	23.48	0.846	0.119
C18:2n-6, Linoleic acid	25.31^a^	21.04^b^	22.56^b^	0.588	0.002
C20:4n-6, Arachidonic acid	7.90	6.61	6.76	0.399	0.379
C18:3n-3, ALA	0.31	0.33	0.32	0.013	0.730
C20:5n-3, EPA	0.08^c^	0.91^a^	0.41^b^	0.094	< 0.001
C22:6n-3, DHA	1.25^c^	9.52^a^	5.64^b^	0.964	< 0.001
SFA	35.70	37.71	38.39	0.586	0.150
MUFA	29.45^a^	23.88^b^	25.92^ab^	0.928	0.031
PUFA	34.85^b^	38.41^a^	35.69^b^	0.556	0.010
n-3 PUFA	1.64^c^	10.76^a^	6.37^b^	1.053	< 0.001
n-6 PUFA	33.21^a^	27.65^b^	29.32^b^	0.715	< 0.001
n-6 /n-3 ratio	22.55^a^	2.66^b^	5.09^b^	2.441	< 0.001

a,b,c Means within row carry on common superscript are significantly different at *P* < 0.05.

1 Each mean represents values from 10 chickens per group (5 replicates × 2 chickens/ replicate).

2 Treatment consisted of 6 % rice bran oil (control), 3 % rice bran oil + 3 % tuna oil (3 % TO), 3 % rice bran oil + 3 % tuna oil in lipid-based nanoparticles (3 % TO-TNP).

3 ALA, Alpha-linolenic acid; EPA, Eicosapentaenoic acid; DHA, Docosahexaenoic acid; SFA, Saturated fatty acids; MUFA, Monounsaturated fatty acids; PUFA, Polyunsaturated fatty acid.

In the thigh meat, both TO groups had lower levels of C18:2n-6, n-6 PUFA, C24:1 and MUFA but higher levels of C14:0, EPA, DHA and n-3 FA than the control group. The n-6/n-3 ratio was 4 to 8 folds lower for the TO groups compared to the control group (*P* < 0.05). The 3 % TO group had higher levels of EPA, DHA, PUFA and n-3 PUFA than the 3 % TO-TNP group (*P* < 0.05) but lower levels of MUFA than the control group (*P* < 0.05). There were no significant differences between groups for the other fatty acids.

### Blood parameters

#### Hematology measurements

The hematology observations of blood samples are presented in Table 8. There was no significant effect of dietary treatments on hematology measurements, except for hemoglobin and hematocrit that were higher in 3 % TO-TNP group compared to the other groups (*P* < 0.05) and the level of white blood cell in TO groups was lower than that of the control group (*P* < 0.05).

**Table 8:** Haematology parameters of Korat chickens fed diets containing rice bran oil (control), tuna oil (3 % TO) or tuna oil in lipid-based nanoparticles (3 % TO-TNP) and measured at 70 days of age^1^.

	Treatments^2^				
Parameters^3^	Control	3 % TO	3 % TO-TNP	SEM	P-value
RBC (x10^6^/mm^3^)	2.23	2.28	2.48	0.06	0.131
Hb (g/dL)	9.46^b^	10.06^b^	11.32^a^	0.27	< 0.05
PCV (%)	28.40^b^	30.20^b^	34.00^a^	0.80	< 0.05
MCV (fL)	13.00	13.50	13.92	0.20	0.171
MCH (Pg)	43.00	44.88	45.88	0.61	0.152
MCHC (g/dL)	33.08	33.10	33.18	0.04	0.619
WBC (x10^6^/mm^3^)	3872^a^	1837^b^	2310^b^	268.90	< 0.05
Heterophils (%)	64.40	55.60	69.40	2.82	0.125
Basophils (%)	3.80	2.80	2.00	0.43	0.253
Eosinophil (%)	1.80	2.00	1.40	0.36	0.809
Lymphocyte (%)	28.20	37.60	25.60	2.66	0.154
Monocytes (%)	1.80	2.00	1.60	0.22	0.790

a,b Means within row carry on common superscript are significantly different at *P* < 0.05.

1 Each mean represents values from 5 chickens per group (5 replicates x 1 chicken/ replicate).

2 Treatment consisted of 6 % rice bran oil (control), 3 % rice bran oil + 3 % tuna oil (3 % TO), 3 % rice bran oil + 3 % tuna oil in lipid-based nanoparticles (3 % TO-TNP).

3 RBC, Red blood cell; Hb, Haemoglobin; PCV, Packed cell volume; MCV, Mean corpuscular volume; MCH, Mean corpuscular haemoglobin; MCHC, Mean corpuscular haemoglobin concentration; WBC, White blood cells.

### Plasma biochemistry

The average values of biochemical parameters measured in plasma are presented in Table 9. Both TO groups had lower HDL concentration than the control group. The 3 % TO-TNP group had lower creatinine plasma concentration but higher alanine amino transferase activity than the control group (*P* < 0.05). The 3 % TO group had a higher aspartate amino transferase activity but a lower uric acid plasma concentration than the other groups (*P* < 0.06). For all the other measured parameters there were no significant differences between groups.

**Table 9:** Biochemical parameters in plasma of Korat chicken fed diets containing rice bran oil (control), tuna oil (3 % TO) or tuna oil in lipid-based nanoparticles (3 % TO-TNP) measured at 70 days of age^1^.

	Treatment^2^				
Parameters^3^	Control	3 % TO	3 % TO_TNP	SEM	P-value
Glucose (mg/dL)	169.60	167.80	157.60	3.41	0.323
Cholesterol (mg/dL)	116.40	101.60	108.00	3.62	0.261
Triglycerides (mg/dL)	50.80^a^	27.40^b^	27.20^b^	4.17	0.015
LDL (mg/dL)	26.40	30.00	28.40	1.03	0.392
HDL (mg/dL)	53.20^a^	45.40^ab^	41.20^b^	1.83	0.011
Total proteins (g/dL)	4.22	4.28	4.16	0.06	0.775
Albumin (g/dL)	2.26	2.40	2.40	0.04	0.275
Total bilirubin (mg/dL)	0.74	0.86	0.96	0.05	0.268
Direct bilirubin (mg/dL)	0.02	0.01	0.02	0.00	0.493
Globulin (g/dL)	1.96	1.88	1.76	0.07	0.486
Blood urea nitrogen (mg/dL)	1.60	2.20	1.80	0.17	0.344
CPK (U/L)	2628.20	2944.40	2453.40	123.67	0.277
LDH (U/L)	845.00	898.00	820.00	33.68	0.661
ALT (U/L)	5.40^b^	6.20^ab^	7.00^a^	0.26	0.029
AST (U/L)	244.20^b^	277.80^a^	246.60^b^	6.66	0.058
Creatinine (mg/dL)	0.42^a^	0.36^ab^	0.32^b^	0.02	0.021
Uric acid (mg/dL)	7.46^a^	4.18^b^	7.18^b^	0.60	0.034
Na (mmol/L)	153.00	154.40	151.20	0.68	0.160
K (mmol/L)	5.30	5.88	5.28	0.13	0.084
Cl (mmol/L)	119.60	118.60	120.20	0.48	0.413
Mg (mg/dL)	2.06	2.14	2.00	0.04	0.331
Ca (mg/dL)	10.20	10.28	10.78	0.17	0.341
Inorganic phosphorus (mg/dL)	8.34	8.42	8.86	0.24	0.663

a,b Means within row carry on common superscript are significantly different at *P* < 0.06.

1 Each mean represents values from 5 chickens per group (5 replicates x 1 chicken/ replicate).

2 Treatment consisted of 6 % rice bran oil (control), 3 % rice bran oil + 3 % tuna oil (3 % TO), 3 % rice bran oil + 3 % tuna oil in lipid-based nanoparticles (3 % TO-TNP).

3 LDL, Low density lipoprotein; HDL, High density lipoprotein; CPK, Creatine phosphokinase; LDH, Lactate dehydrogenase; ALT, Alanine amino transferase; AST, Aspartate amino transferase; Na, Sodium; K, Potassium; Cl, Chloride; Mg, Magnesium; Ca, Calcium.

## Discussion

### Enrichment with n-3 PUFA of slow-growing chicken meat with targeted lipid nanoparticles

The first aim of the study was to test the possibility of enriching chicken meat with tuna oil embedded in targeted lipid nanoparticles. The accumulation of n-3 PUFAs in chicken meat through lipid nanoparticles supports our hypothesis that these fatty acids, when delivered via targeted nanotechnology, are effectively deposited in the meat of slow-growing chickens. This is evidenced by a 3.5- and 3.9-fold increase in n-3 PUFA content in breast and thigh meat, respectively, in the 3 % TO-TNP group compared to the control group. Additionally, the n-6/n-3 ratio was reduced by a factor of 4.0 and 4.4 in the breast and thigh meat, respectively. These present findings align with those of Rymer et al. (2010) and Abbasi et al. (2019), who used microencapsulation and nanoemulsions, respectively.

Although n-3 PUFA-enriched meat was successfully produced by supplementing the diet with 3 % TO-TNP, the accumulation of these fatty acids in the meat was still lower than in the 3 % TO group. Specifically, the n-3 PUFA content in both breast and thigh meat were 1.4- to 1.5-fold lower in the 3 % TO-TNP group, and DHA accumulation was 1.7-fold lower. This discrepancy is primarily attributed to the lower initial levels of dietary n-3 PUFAs and DHA in the 3 % TO-TNP group, which were 2.4-fold lower for n-3 PUFA and 2.8-fold lower for DHA compared to the 3 % TO group. The reduced content of n-3 PUFAs in the lipid nanoparticles likely resulted from processing conditions, such as the material ratio used during synthesis and the heating step, both of which are known to affect the entrapment efficiency of nanoparticles (Pang et al., 2017; Venugopalan et al., 2021). These factors led to a reduction in accumulation of n-3 PUFA and DHA in the meat compared to crude tuna oil.

Compared to Hang et al. (2018a), who reported that supplementing slow-growing Korat chickens with 2 % and 4 % tuna oil resulted in n-3 PUFA and DHA levels being twofold lower in the 2 % tuna oil diet than in the 4 % tuna oil diet, our study presents an interesting contrast. Hang et al. (2018a) found that this dietary difference led to a 1.4- and 1.8-fold reduction in n-3 PUFA content in breast and thigh meat, respectively, while DHA accumulation decreased by 1.5- and 2.1-fold in the 2 % tuna oil group. In contrast, despite the dietary n-3 PUFA and DHA content in the 3 % TO-TNP group being 2.4- to 2.8-fold lower than in the 3 % TO group, our study found that their accumulation in breast and thigh meat was only about 1.4- to 1.7-fold lower. This suggests that lipid nanoparticles may enhance the deposition efficiency of n-3 PUFAs, potentially improving their bioavailability.

The ability of the 3 % TO-TNP group to maintain high accumulation efficiency despite lower dietary n-3 PUFA levels may be due to the nanoparticle delivery system, which could help preserve n-3 PUFA and DHA stability while enhancing their bioavailability (Yeh et al., 2014; Kou et al., 2017; Tian et al., 2017; Yang et al., 2019). However, increased nanoparticle uptake may also trigger metabolic conversion processes, potentially reducing n-3 PUFA and DHA levels in meat. Excessive n-3 PUFA intake in muscle cells has been reported to influence β-oxidation (fat breakdown), shifting metabolism from anaerobic to aerobic, increasing ATP production, and improving overall energy balance for muscle function (Stupin et al., 2019). This metabolic shift may explain the reduction in n-3 PUFA and DHA levels observed in the 3 % TO-TNP group, in addition to the initially lower levels of n-3 PUFA. Supporting our hypothesis, the elevated hemoglobin and hematocrit levels in this group suggest a potential shift in metabolic conversion, leading to molecular-level changes that influence n-3 PUFA deposition. To better understand these mechanisms and their interconnections, further investigations should explore metabolic pathways related to hemoglobin regulation and lipid metabolism, potentially through targeted biomarker analysis or proteomic profiling.

Additionally, although no differences were observed in the fatty acid composition of breast and thigh meat between the two TO groups—including myristic, palmitic, stearic, oleic, nervonic, linoleic, linolenic, and arachidonic acids —the myristic acid level in meat from the TO groups was higher than that in the control diet. This increase was likely due to the higher myristic acid content in the fish oil-based diet (Al-Khalaifah et al., 2020). The TO groups also had a lower content of nervonic acid in breast and thigh meat, suggesting a less active synthesis from its precursor (C18:1n-9; Liu et al., 2021) compared to the control group. Moreover, the levels of linoleic, and arachidonic acids in meat from the TO groups were lower than those in the control diet, which could be attributed to the fatty acid composition of the rice bran oil-based diet (Hang et al., 2018a).

It can be concluded that while these findings highlight the promise of nanotechnology in functional meat production, the effectiveness of lipid nanoparticles remains constrained by the initially low levels of fatty acids within them. This limitation emphasizes the need for further optimization of the synthesis process to improve fatty acid entrapment and maximize n-3 PUFA enrichment in poultry meat. By refining these nanoparticle formulations, it may be possible to develop more efficient strategies for producing n-3 PUFA-enriched meat while minimizing dietary inclusion levels.

### Evaluation of the safety of targeted lipid nanoparticles

The second aim of this study was to evaluate the potential effects of lipid nanoparticles on various indicators of chicken growth performance and health. In this study, all experimental diets were formulated to contain equal metabolizable energy (ME) and crude protein (CP) levels across all treatment groups, ensuring uniform nutrient availability. This dietary uniformity may have contributed to the absence of significant differences observed in growth performance, mortality rate, or carcass composition. Additionally, the relatively short feeding period of only 21 days may not have been sufficient to detect measurable effects on these parameters. These findings are consistent with previous studies by Chen et al. (2012); Hang et al. (2018b), and Khosinklang et al. (2023). However, several factors may contribute to variations in growth performance, particularly the duration of supplementation. For instance, initiating supplementation from the first day has been reported to enhance final body weight (López-Ferrer et al., 2001). Moreover, the type of oil used in combination with fish oil, such as poultry fat (Farhoomand and Checaniazer, 2009), tallow (López-Ferrer et al., 2001), and soybean oil (Abbasi et al., 2019), may influence growth responses. The balance of the fatty acid profile is also a key factor in determining whether growth is enhanced or remains unchanged (Poorghasemi et al., 2013).

The macroscopic examination revealed no adverse effects on the weights of the heart, spleen, liver, abdominal fat, muscles, or meat characteristics among the treatment groups. However, the higher gizzard percentage relative to hot carcass weight observed in both TO groups, as also reported by Avi et al. (2023), suggests that n-3 PUFA supplementation may contribute to improved carcass characteristics. Moreover, it did not exhibit any adverse effects on the quality traits of breast and thigh meat.

Regarding plasma concentrations of cholesterol and LDL in this study, no significant differences were observed between the groups. However, plasma triglyceride and HDL concentrations were lower in the TO groups than in the control group. These results contrast with the findings of Ibrahim et al. (2018), Abbasi et al. (2019), and Avi et al. (2023), who reported that cholesterol and triglyceride levels decreased while HDL levels increased with higher consumption of oils rich in n-3 PUFAs, particularly EPA and DHA. The increase in HDL and triglyceride levels in the control group in this study may be attributed to its high content of oleic and linoleic acids, as reported by Viveros et al. (2009) and Ibrahim et al. (2018). The reduction in plasma HDL levels in both TO groups is likely explained by the major role of n-3 PUFAs as agonists of peroxisome proliferator-activated receptor-alpha (PPARα). Activation of PPARα upregulates the expression of cholesteryl ester transfer protein (CETP), facilitating the transfer of cholesteryl esters from HDL to other lipoproteins. This triglyceride-enriched HDL particles becoming susceptible to hydrolysis by hepatic lipase led to increasing HDL clearance from circulation (Raposo et al., 2014). Although a reduction in HDL levels may appear unfavorable, supplementation with EPA and DHA-rich sources has been shown to reduce triglyceride accumulation while potentially enhancing cholesterol clearance. This process may help prevent excessive cholesterol buildup, which is associated with cardiovascular diseases (Sherratt et al., 2023). Thus, these findings suggest that the TO groups contribute to improved cholesterol homeostasis and support a cardioprotective effect.

Interestingly, the supplementation of tuna oil in the form of nanoparticles may also contribute to this effect, as it appeared to reduce the HDL to the same level as the normal form of tuna oil. In general, while mammals absorb dietary lipid nanoparticles primarily through the lymphatic system, poultry predominantly absorb them via the portal-mesenteric system, enabling direct transport to the liver for metabolic processing (Ali Khan et al., 2013; Ravindran et al., 2016). Hypothetically, the targeted lipid nanoparticles are composed of glucose and have the potential to be absorbed by enterocyte cells via glucose transporters, thereby enhancing absorption and increasing their entry into the circulation (Wangngae et al., 2023). Thus, alternative absorption pathways of lipid-based nanoparticles by enhancing first-pass liver metabolism, may improve efficiency in activating HDL clearance, reaching levels equivalent to the 3 % TO group.

More importantly, the high absorption efficiency and rapid metabolism of n-3 PUFAs may indirectly contribute to hemolysis, ultimately leading to alterations in blood chemistry and hematological parameters, including elevated LDH levels (Kato et al., 2006), increased total protein, and decreased albumin levels (Roman et al., 2009). In this study, no significant differences were observed in hematological parameters among the groups, suggesting a lack of hemolysis or potential toxicity from tuna oil supplementation in either forms. While hemolysis or other signs of blood-related stress were not influenced by our treatment groups, the results also revealed the highest hemoglobin and hematocrit levels in chickens fed the 3 % TO-TNP diet. Excessive intake of n-3 PUFAs or metabolic stress has been shown to induce oxygen deficiency by increasing the rate of fatty acid metabolism to generate more energy, thereby elevating cellular oxygen demand (Winter et al., 2016; de la Harpe et al., 2019). Thus, higher hemoglobin and hematocrit levels may enhance the blood's oxygen-carrying capacity, facilitating better oxygen delivery to tissues. Moreover, the lower white blood cell counts observed in both TO groups could be attributed to the well-established anti-inflammatory effects of n-3 PUFAs, which reduce pro-inflammatory cytokine production, promote macrophage polarization toward an anti-inflammatory phenotype, and enhance the resolution of inflammation (Al-Khalifa et al., 2012). These findings further emphasize the safety of lipid nanoparticles in this study, highlighting the potential metabolic effects of using n-3 PUFAs in lipid nanoparticle form.

The blood biochemical parameters of the present study were within the standard ranges of chickens as reported by Abdulazeez et al. (2016) and Soualio et al. (2021)*.* The creatine phosphokinase present in the blood can serve as an indicator of liver injury (Navidshad et al., 2019) and muscle tissue activity (Baird et al., 2012). Therefore, the absence of significant differences among treatment groups suggests that the dietary interventions did not negatively impact liver or muscle function. Additionally, AST, ALT, uric acid, and creatinine serve as key indicators of liver and renal function, particularly in toxicity assessments (Fassett et al., 2011; Mohammadi et al., 2016). Both TO groups exhibited higher ALT activity than the control group, and 3 % TO group had higher AST activity than the other groups. Comparing different oil sources in diets, Elbaz et al. (2023) also reported higher ALT and AST activities in plasma of broiler chickens fed diet containing 3 % fish oil. This may suggest that TO induced higher lipid peroxidation activity in the liver stimulating ALT and AST activities. Nevertheless, the ALT and AST activities remained within the normal ranges for chickens (Gudiso et al., 2019). Interestingly, previous study has shown that uric acid plays an important role as an endogenous antioxidant in chickens by neutralizing free radicals and reactive oxygen species (ROS), thereby protecting cells against oxidative stress (Tantiyasawasdikul et al., 2023). It is possible that the reduced level of uric acid may be associated with an increased demand for antioxidant responses due to the excessive intake of n-3 PUFAs in the 3 % TO supplemented group, which provides the highest levels of EPA and DHA, as these two fatty acids are highly susceptible to oxidation. Creatinine levels indicate kidney function, with higher concentrations signifying impairment (Fitri et al., 2021). EPA and DHA supplementation can enhance renal function by mitigating decline and reducing hypertension (Noor et al., 2023). The lower creatinine levels in the n-3 PUFA-enriched diet suggest improved kidney function, likely through reducing pro-inflammatory lipid mediators (Rund et al., 2020). This implies that both TO groups similarly slowed renal function loss. Finally, no significant differences were observed between groups for other plasma biochemical parameters measured.

### Optimizing n-3 PUFA-lipid nanoparticle for enhanced bioavailability in chicken nutrition

This study highlights the need to enhance lipid nanoparticle formulation to maximize n-3 PUFA encapsulation and minimize losses, ultimately improving efficiency in chicken nutrition. Although tuna oil-loaded lipid nanoparticles (TO-TNP) successfully increased n-3 PUFA content in chicken meat, encapsulation efficiency and lipid stability remain key challenges. Thermal processing and lipid matrix composition influence nanoparticle integrity, affecting PUFA retention and bioavailability (Pang et al., 2017; Venugopalan et al., 2021). Future improvements should focus on enhancing encapsulation techniques, such as optimizing homogenization intensity, emulsifier selection, and drying methods, to protect PUFAs from degradation (Yaghmur et al., 2023). A more stable formulation would allow for lower supplementation levels while maintaining high deposition efficiency, reducing dietary requirements and improving cost-effectiveness. Refining lipid nanoparticle technology offers a promising pathway for enhancing n-3 PUFA deposition in poultry meat, ensuring greater nutritional benefits with lower supplementation levels. Further research should focus on improving nanoparticle stability and absorption efficiency, making n-3 PUFA delivery more effective in chicken production.

## Conclusions

This research is the first demonstration that it is possible to enrich the meat of livestock with n-3 PUFA by using diets containing targeted lipid nanoparticles made with tuna oil. In the present study, the enrichment with n-3 PUFA was less efficient than that obtained with crude tuna oil directly incorporated in the diet. Further research is needed to optimize this synthesis process but also to understand how the targeted lipid nanoparticles cross the intestinal epithelium barrier, are transported into the blood and/or lymphatic circulation and how the n-3 PUFA are delivered into the muscles. The present study also showed that targeted lipid nanoparticles had no negative effects on liver, muscles and kidney functions. Nevertheless, as the final consumer of chicken meat is human, the safety of poultry meat enriched with n-3 PUFA lipid nanoparticles should be controlled. Finally, when the synthesis process of targeted lipid nanoparticles will be stabilized, it will be interesting to realize an economical evaluation of the feed cost.

## Disclosures

The authors declare no conflict of interest.
